# Diagnostic Performance of a Rapid Magnetic Resonance Imaging Method of Measuring Hepatic Steatosis

**DOI:** 10.1371/journal.pone.0059287

**Published:** 2013-03-21

**Authors:** Michael J. House, Eng K. Gan, Leon A. Adams, Oyekoya T. Ayonrinde, Sander J. Bangma, Prithi S. Bhathal, John K. Olynyk, Tim G. St. Pierre

**Affiliations:** 1 School of Physics, The University of Western Australia, Crawley, Australia; 2 School of Medicine and Pharmacology, The University of Western Australia, Crawley, Australia; 3 Department of Gastroenterology, Fremantle Hospital, Fremantle, Australia; 4 Liver Transplant Unit, Sir Charles Gairdner Hospital, Nedlands, Australia; 5 Resonance Health Ltd, Claremont, Australia; 6 Department of Pathology, The University of Melbourne, Melbourne, Australia; 7 Curtin Health Innovation Research Institute, Curtin University of Technology, Bentley, Australia; 8 Institute for Immunology & Infectious Diseases, Murdoch University, Murdoch, Australia; Copenhagen University Hospital Gentofte, Denmark

## Abstract

**Objectives:**

Hepatic steatosis is associated with an increased risk of developing serious liver disease and other clinical sequelae of the metabolic syndrome. However, visual estimates of steatosis from histological sections of biopsy samples are subjective and reliant on an invasive procedure with associated risks. The aim of this study was to test the ability of a rapid, routinely available, magnetic resonance imaging (MRI) method to diagnose clinically relevant grades of hepatic steatosis in a cohort of patients with diverse liver diseases.

**Materials and Methods:**

Fifty-nine patients with a range of liver diseases underwent liver biopsy and MRI. Hepatic steatosis was quantified firstly using an opposed-phase, in-phase gradient echo, single breath-hold MRI methodology and secondly, using liver biopsy with visual estimation by a histopathologist and by computer-assisted morphometric image analysis. The area under the receiver operating characteristic (ROC) curve was used to assess the diagnostic performance of the MRI method against the biopsy observations.

**Results:**

The MRI approach had high sensitivity and specificity at all hepatic steatosis thresholds. Areas under ROC curves were 0.962, 0.993, and 0.972 at thresholds of 5%, 33%, and 66% liver fat, respectively. MRI measurements were strongly associated with visual (r^2^ = 0.83) and computer-assisted morphometric (r^2^ = 0.84) estimates of hepatic steatosis from histological specimens.

**Conclusions:**

This MRI approach, using a conventional, rapid, gradient echo method, has high sensitivity and specificity for diagnosing liver fat at all grades of steatosis in a cohort with a range of liver diseases.

## Introduction

Fatty liver disease is associated with an increased risk of carcinoma, cardiovascular death, cirrhosis, reduced effectiveness of antiviral treatments, and is implicated in the development of diabetes [1]. A visual estimate of the fat content in a liver biopsy specimen by a histopathologist is considered the gold standard for clinically assessing liver fat. Liver fat levels are generally estimated by the percentage of hepatocytes containing intracellular fat vacuoles graded categorically on a scale from 0 to 3 [2] or on a continuous scale from 0 to 100%. Visual estimates of liver fat in biopsy samples are subjective, have poor reproducibility [3], are potentially unrepresentative of the whole liver and require an invasive procedure with associated risks to obtain the sample [4]. Some patients with fatty liver disease are biopsied as part of their routine clinical assessment to determine the severity of fibrosis or inflammation. However, with obesity now common in developed countries there is increased interest in non-invasive methods of quantifying liver fat content for research purposes, diagnosis, and monitoring intervention programs.

Magnetic resonance imaging (MRI) and spectroscopy (MRS) have been used for decades to qualitatively and quantitatively assess liver fat [5], [6]. Spectroscopy is currently considered the most accurate quantitative MR method for measuring liver fat, but imaging approaches based on the Dixon method offer the advantage of assessing larger regions of the liver in a comparatively short time and with simpler image processing. Furthermore, use of MRS is largely limited to research settings due to the technical expertise and cost required to routinely deliver it.

While several reports have compared histological liver fat estimates with quantitative MRI approaches [7]–[25], there are fewer studies [7], [9], [15], [22], [23], [26], [27] which have assessed the diagnostic performance of MRI using a pathologist’s visual estimate of fat content as the reference standard to identify the clinically relevant thresholds as defined by the Nonalcoholic Steatosis Clinical Research Network (NASH CRN) [2], [28]. The largest of these diagnostic studies [15] investigated a cohort of potential living donors without any documented liver disease. Some studies have also supplemented the histopathologist’s visual estimate of fat with computer assisted image processing techniques to objectively measure the area and hence volume fraction occupied by vesicular fat in the biopsy histological section. These image-processing approaches generally use either stereology counting [20], [21] or image segmentation [16] with shape analysis [18], [19], [29] to quantify the area of fat in a biopsy sample. Only two studies [18], [29] have compared a quantitative MRI method to fat measurements based on a histopathologist’s visual estimate and those based on morphometric image analysis of the biopsy histological section.

The general approach for measuring liver fat using MRI is to reduce or correct for confounding factors so that there is, as close as possible, a linear, one-to-one relationship between the measured MRI signal and the volume fraction of liver fat. While potentially improving the direct relationship between the MRI signal and liver fat, the acquisition adjustments to minimise confounding factors lead to poor signal to noise in the acquired images, reducing the generic diagnostic quality and potentially the diagnostic sensitivity. Rather than attempt to obtain a measured signal that accurately represented the proton density ratio of fat to water, we took a calibration-based approach that purposefully distorted the measured signal away from the fat-water proton density ratio in order to increase the sensitivity of the measurement to the presence of fat and maintain diagnostic image quality. Hence, the primary aim of this study was to test the ability of this approach, which comprised a triple gradient echo, single breath-hold MRI method, to diagnose clinically relevant grades of hepatic steatosis in a diverse cohort using a histopathologist’s visual estimate of fat content as the reference standard according to the NASH CRN thresholds [2], [28]. A secondary aim was to measure the diagnostic performance of MRI to grade liver fat when a reference standard based on quantitative computer assisted morphometric image analysis of histological sections was used instead of a histopathologist’s visual assessment.

## Materials and Methods

### Ethics Statement

Written informed consent was obtained from each subject and the study protocol conformed to the ethical guidelines of the 1975 Declaration of Helsinki as reflected in approval by the Fremantle Hospital Human Research Ethics Committee and the Sir Charles Gairdner Hospital Human Research Ethics Committee.

### Subjects

Ten healthy controls and 65 patients were enrolled in the study. The patients were recruited from the hepatology outpatient clinics at Fremantle and Sir Charles Gairdner Hospitals, Western Australia. The patient inclusion criteria were: age between 18 and 75 years and written informed consent. Control subjects were not biopsied and required a body mass index (BMI) less than 25 and no history of liver disease. The control subjects were included to provide a baseline of normal liver fat levels as measured by this MRI technique. Exclusion criteria were: contraindications for MRI, pregnancy or lactation. One patient was also excluded after their history indicated fluctuations in weight and alcohol consumption during the study period. An additional five cases were excluded for incorrect MRI acquisition or unavailability of images for morphometric analysis leaving 59 patients that entered the study (Table S1 in File S1).

### Liver Histology and Quantification of Liver Fat

The patients underwent percutaneous liver biopsy with ultrasound guidance as part of their routine clinical management. Each liver biopsy specimen was reviewed by an experienced hepatopathologist (PB), blinded to the patient's identity and MRI data, who visually estimated the percentage of hepatocytes containing fat on a continuous scale between 0% and 100% (HIS-VIS). The METAVIR scoring system was used to stage the amount of fibrosis.

### Computer Assisted Morphometric Image Analysis of Liver Fat Area

**Figure 1:**
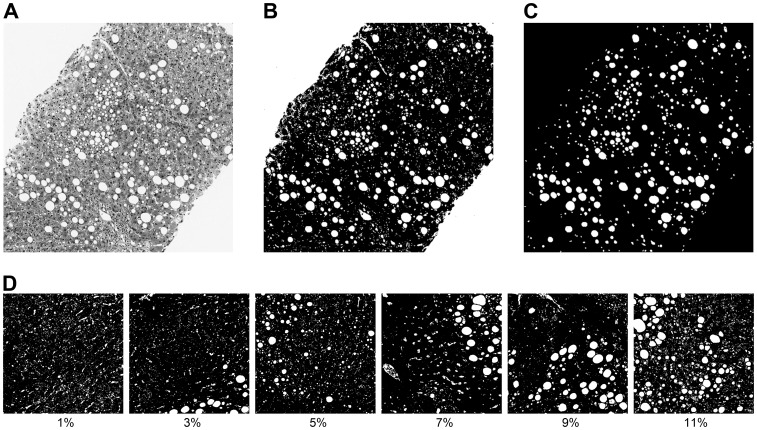
Example of histology images and morphometric image analysis. A) Histologic image of a liver (Masson trichrome, ×20 objective), B) Binary image of same image after application of threshold, C) Mask showing fat vacuoles after application of size and structural criteria, D) Examples of binary histology images with measured fat percentage areas. These images have been thresholded as in 1B, but not masked (as in 1C), so as to keep the additional white spaces that are not represented in the areal fat estimate, but are visible in a histology image. Each square is 500 microns across.

The fraction of vesicular fat vacuoles in Masson’s trichrome stained histological sections was assessed by computer assisted morphometric image analysis. Histological sections of the biopsies were scanned in colour using an Aperio Scanscope XT (Aperio Technologies, Inc., California, USA) automated slide scanner and ImageScope software. Using ImageJ 1.42 (NIH, USA) software, the fat vacuoles were automatically identified and areas measured in a multi-stage process using thresholding, size, and circularity criteria. Fat vacuoles, holes, tears or vessels in the biopsy section appear as high intensity (white) areas (Figure 1A). These high intensity regions were automatically segmented by applying a threshold intensity of 220 (out of 256) on the green image band, which on inspection provided the best contrast between fat vacuoles and liver parenchyma. The threshold image was converted into a binary image such that the fat vacuoles and any other high intensity regions were given a value of 255 and non-fat tissue a value of 0 (Figure 1B). The fat vacuoles were then automatically identified and delineated using the Analyze Particles tool in ImageJ with a circularity index between 0.5 and 1 and a size threshold between 100 and 10000 pixels (equivalent to diameters from 5.6 to 56 µm). The circularity index ranges from 0 (infinitely elongated polygon) to 1 (perfect circle) and is defined as (4π × Area)/Perimeter^2^.

The criteria for thresholding intensity, size, and circularity were established by inspection of the results of different thresholds, sizes and circularities on the effective simultaneous exclusion of large vessels, ducts or other large areas of high intensity and inclusion of fat vacuoles. The analysis produced the size and circularity of each individual fat vacuole and the total area of all the fat vacuoles within the threshold ranges. To compute the total area of the biopsy sample, the binary image was reversed and the Fill Holes tool used to produce a biopsy image without holes. The total area of the biopsy sample was measured and the areal fat fraction (HIS-MORPH) computed from the ratio of fat area to total biopsy tissue area.

### MRI

#### Data acquisition

All MRI measurements were made on Siemens 1.5 T Avanto scanners (Siemens Medical Systems, Erlangen, Germany) at Fremantle Hospital, St John of God Murdoch Hospital, and Hollywood Private Hospital, Western Australia. The median time between biopsy and MRI was 57 days. Phased-array torso coils were centred over the liver of the subjects. MRI acquisition comprised an opposed-phase, in-phase, opposed-phase gradient echo sequence (TEs 2.38, 4.76, 7.14 ms, TR 88 ms, 1 excitation, flip angle 70 degrees, bandwidth 500 Hz). Data from three axial slices, positioned through the widest part of the liver, were acquired in a single breath-hold. The slice thickness was 4 mm and the matrix was 256×256 with a field of view 300×300 mm. Liver iron concentrations (LIC) were measured using a validated non-invasive MRI method (FerriScan®) [30]–[32].

#### Image processing

**Figure 2:**
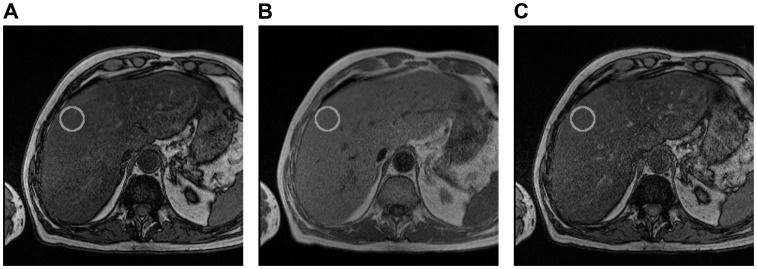
Axial magnetic resonance images of a patient with a typical ROI (solid grey circle) used for fat analysis. A) 2.38 ms (opposed phase), B) 4.76 ms (in phase), C) 7.14 ms (opposed phase).

A single analyst (MJH), blinded to the identity and medical histories of the subjects, reviewed and processed all images using ImageJ 1.42 (NIH, USA). On each of the three slices a circular region of interest (ROI) about 580 mm^2^ was delineated within the right lobe of the liver, avoiding large intrahepatic vessels and any obvious motion-affected regions (Figure 2). The average image intensities within the ROIs, for all three echoes and slices, were used to calculate a parameter, α, (Eqn. 1). The final value of α reported was the average for the three slices. The parameter α can take any value between 0 and 1 and is related to the liver fat concentration, but is also dependent on the MRI pulse sequence parameters.

The parameter α in the liver ROI was calculated for each slice using the following equation:
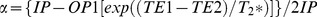
(1)where IP is image intensity in the ROI of the first in-phase image, OP1 is image intensity in the ROI of the first opposed-phase image, TE1 is the first opposed-phase echo time, and TE2 is the in-phase echo time [33]. A T_2_^*^ estimate for correcting the T_2_^*^ signal decay of the OP1 echo was derived from the two OP images using the equation:
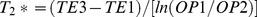
(2)where OP1 and OP2 are the signal intensities in the ROI of the first and second opposed phase images, TE1 is the first opposed-phase echo time, and TE3 is the second opposed-phase echo time.

#### Analysis

The relationship between α and the volume fraction of fat in the liver is not expected to be linear owing to both the difference in proton density between fat and the surrounding tissue and the difference in T_1_ (the MRI related parameter known as the longitudinal relaxation time constant) between fat and the surrounding tissue. The relationship between α and volume fraction (*f*) of fat in the liver is expected to be of the form.
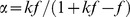
(3)where *k* is a constant [33]. Since the fat vacuoles are approximately spherical and the histological sections are thin compared to the size of the fat vacuoles, the area fraction of the histological section accounted for by fat vacuoles (HIS-MORPH) will be equal to the volume fraction of fat in the liver tissue.

### Statistical Analysis

Descriptive clinical and demographic characteristics were compared using Chi-squared analysis (for categorical data), the Student’s *t* test (for continuous parametric data), or the Mann-Whitney test (for non-parametric data). Data were tested for normality using the Komolgorov-Smirnov test. Non-Gaussian distributions were summarised by their median value and range. For non-Gaussian distributions the 95% prediction interval was calculated on the log-transformed data. Relationships between continuous parameters were assessed using the coefficient of determination (r^2^). The performance of the MRI technique for predicting the histologically measured fat grades was assessed using receiver operating characteristic (ROC) curve analysis. The area under the ROC curve (AUC) was used to assess the diagnostic performance of the MRI method against the biopsy observations. The thresholds of α were identified by the cut-off that produced the highest combined sum of sensitivity and specificity for distinguishing histological fat scores above and below the standard thresholds. Two ROC curve analyses were performed. The first analysis used the NASH CRN histological scoring system cut-off values of ≥5%, >33%, and >66% liver fat [2], as determined by the visual estimation of the hepatopathologist (PB), to define the diagnostic groups above and below the three cut-offs. A second ROC curve analysis used cut-offs in HIS-MORPH fractions of ≥0.014, >0.043, and >0.077. These morphometric cut-offs were derived from the regression line of HIS-MORPH against HIS-VIS using the visual histopathologist cut-offs of 5%, 33% and 66% as input into the regression equation. The methods of Bland and Altman [34] were used to assess the 95% limits of agreement between MRI and biopsy (HIST-MORPH) fat estimates. A p value less than 0.05 was considered statistically significant.

## Results

### Demographic and Clinical Data

The control subjects were younger and had lower BMIs than the patients (Table 1). BMI values were normally distributed. Thirty-two patients had no/mild fibrosis (METAVIR 0 or 1) and 27 patients had moderate/severe fibrosis (METAVIR 2, 3 or 4). Five patients had liver iron concentration levels above the normal maximum level of 1.8 mg[Fe]/g dry tissue. The median liver fat level of the 59 patients was 5% (range 0 to 98%) from HIS-VIS and 0.029 (range 0.002 to 0.216) from HIS-MORPH. The median value of α for the control subjects (0.024, range 0.013 to 0.105) was significantly lower compared with the patients (0.072, range 0.01 to 0.41, p = 0.012). The 95% prediction interval of α for the control subjects was 0.008 to 0.094. This prediction interval can be viewed as an estimate of the reference range of MRI α values for healthy subjects without liver problems. Interestingly, one of the ten control subjects without any recognised liver condition was outside this reference range.

**Table 1:** Clinical data of the study cohort.

Characteristics	Controls	Chronic Liver Disease Patients	Patients with MRI and Morphometry
N	10	65	59
Gender (female/male)	3/7	31/34	29/30
Age (years), median (range)	33.5 (24–47)	56 (20–72)*	56 (20–72)*
BMI (kg/m^2^), mean ± st. dev.	22.55±1.77	29.00±5.11#	28.92±5.17#
LIC (mg/g), median (range)	1.2 (0.4–1.8)	0.9 (0.3–4.8)	0.9 (0.3–4.4)
Diagnosis			
AIH		3	3
ALD		3	2
HBV-HCV		18	16
NAFLD		11	10
NASH		19	17
NORM		3	3
PSC		4	4
OTHER		4	4

* Significantly different from controls, p<0.05 Mann-Whitney test.

# Significantly different from controls, p<0.05 unpaired t-test. Abbreviations: AIH, autoimmune hepatitis; ALD, alcoholic liver disease; BMI, body mass index; HBV-HCV, chronic viral hepatitis B/C; LIC, liver iron concentration; MRI, magnetic resonance imaging; NAFLD, non-alcoholic fatty liver disease – simple steatosis; NASH, nonalcoholic steatohepatitis; NORM, normal; PSC, primary sclerosing cholangitis.

### Performance of MRI for Predicting Histological Fat Levels

The ROC curve analyses are summarised in Tables 2 and 3. The analyses showed that the MRI α values had high AUCs, sensitivities and specificities at all three liver fat thresholds (Table 2, 3). Based on the ROC curve analysis, the clinically relevant NASH CRN steatosis grade boundaries of 5%, 33% and 66% correspond to MRI α values of 0.067, 0.135, and 0.171, respectively (Table 2). There were no significant differences between the AUC’s from morphometric image analysis compared with the pathologist’s visual estimate (Table 2, 3).

**Table 2:** Analysis of the area under the receiver operating characteristic curve using the histopathologist’s visual estimate of fat in the histological sections.

Cut-off	MRI Cut off Value (α)	AUC	p value	Sensitivity	95% CI	Specificity	95% CI
≥5% (Grade 0 vs 1–3)*	>0.067	0.9615	<0.0001	90.91	75.67 to 98.08	96.15	80.36 to 99.90
>33% (Grade 0–1 vs 2–3)*	>0.135	0.9928	<0.0001	100.0	85.18 to 100.0	97.22	85.47 to 99.93
>66% (Grade 0–2 vs 3)*	>0.171	0.9724	<0.0001	100.0	79.41 to 100.0	88.37	74.92 to 96.11

* Cut-offs are defined according to the NASH CRN grading system. Abbreviations: AUC, Area under receiver operating characteristic curve; CI, confidence interval; MRI, magnetic resonance imaging.

**Table 3:** Analysis of the area under the receiver operating characteristic curve using the morphometric image analysis estimate of fat in the histological sections.

Cut-off	MRI Cut off Value (α)	AUC	p value	Sensitivity	95% CI	Specificity	95% CI
≥0.014	>0.060	0.9639	<0.0001	90.91	75.67 to 98.08	96.15	80.36 to 99.90
>0.043	>0.141	0.9925	<0.0001	100.0	83.89 to 100.0	92.11	78.62 to 98.34
>0.077	>0.188	0.9869	<0.0001	100.0	79.41 to 100.0	93.02	80.94 to 98.54

Abbreviations: AUC, Area under receiver operating characteristic curve; CI, confidence interval; MRI, magnetic resonance imaging.

### Relationship between MRI and Histology

**Figure 3:**
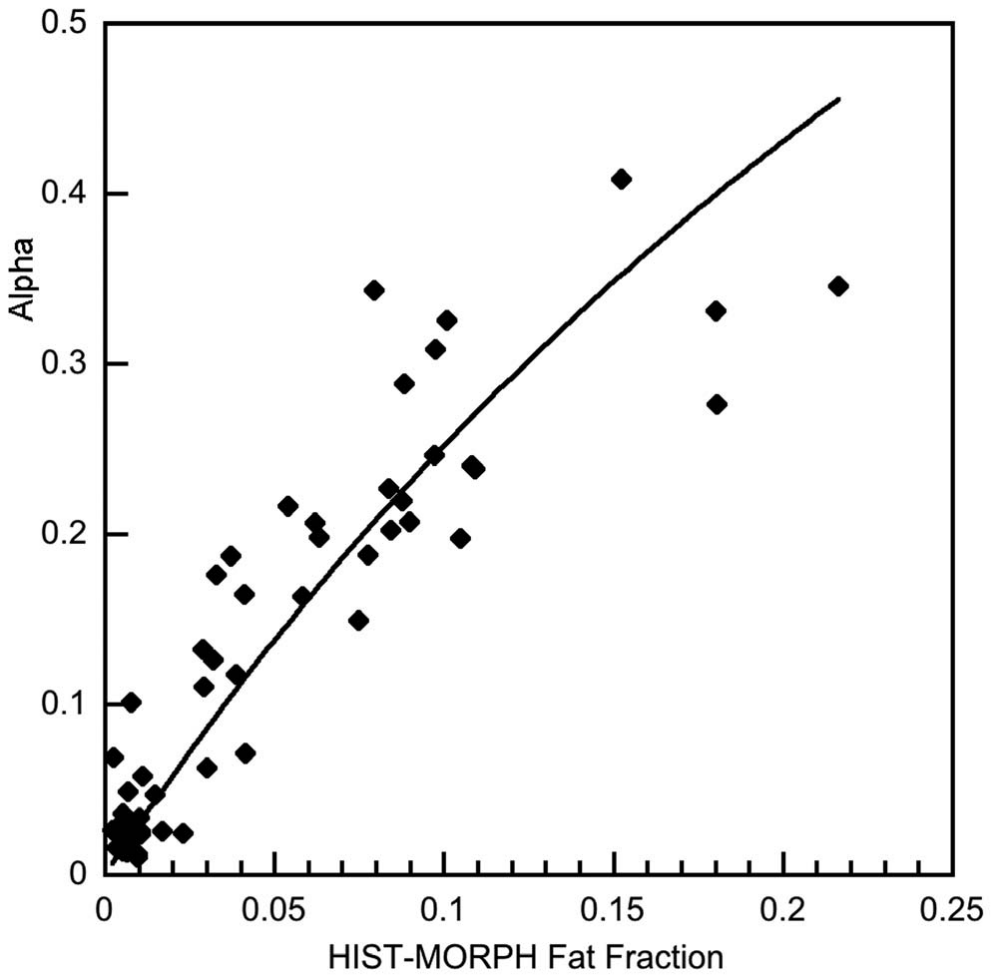
Plot of the MRI derived α value versus the fractional area of fat vacuoles in the histological section (HIST-MORPH). The solid line is a fit of Equation 3 to the data (r^2^ = 0.84).

**Figure 4:**
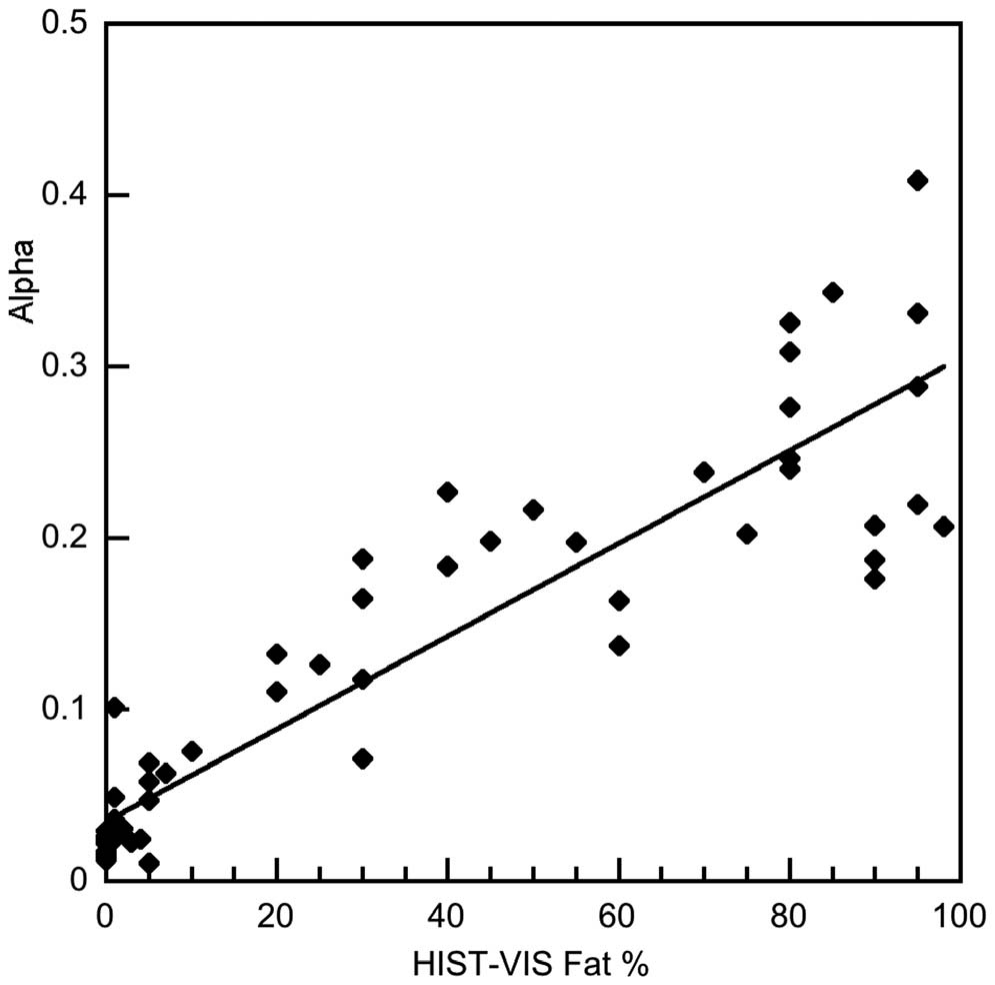
Plot of the MRI derived α value versus histopathologist’s estimate of percentage of hepatocytes containing a fat vacuole (HIST-VIS). The solid line is the linear regression fit to the data (r^2^ = 0.83).

Figure 3 shows a fit of Equation 3 to the α versus HIS-MORPH data. The coefficient of determination, r^2^, for this fit was 0.84. Linear regression analysis showed that there were significant coefficients of determination between the pathologist’s visual estimates of fat (HIST-VIS) and α from the MRI measurement (r^2^ = 0.83, Fig. 4) and between the pathologist’s visual estimates of fat (HIST-VIS) and the morphometric fat fraction HIS-MORPH (r^2^ = 0.72).

### Limits of Agreement between Liver Fat Measurements

**Figure 5:**
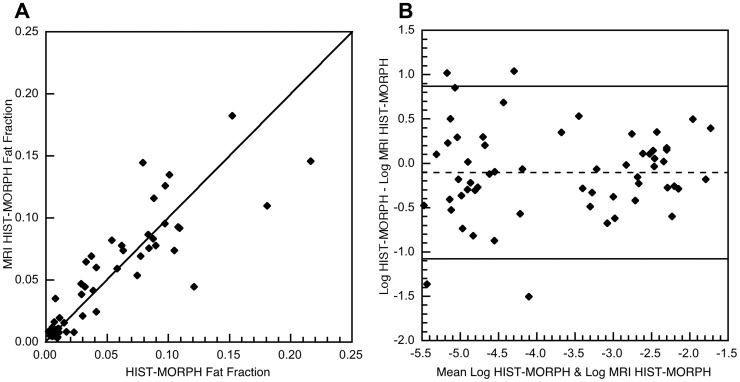
Limits of agreement between liver fat fraction measurements. A) MRI HIST-MORPH fat fraction plotted against HIST-MORPH fat fraction for the 59 subjects. The solid line is the line of equivalence. B) The difference between the natural logarithm of HIST-MORPH and the natural logarithm of MRI HIST-MORPH plotted against the mean of the two logarithms for the 59 subjects. The solid lines indicate the 95% limits of agreement and the dashed line is the mean difference between the logarithms of the two methods.

The MRI alpha results were transformed to equivalent HIST-MORPH values using Eqn. [3] and these values (MRI HIST-MORPH fat fraction) are plotted against the HIST-MORPH measurements (Figure 5a). A plot of the difference against the mean of the two measurements indicated that there was an increase in the variance of the difference between the measurements as the magnitude increased. Hence, the measurements were not amenable to Bland and Altman [34] analysis without transformation. The natural logarithms of each measurement were therefore calculated and the difference between the natural logarithms of the two fat measurements plotted against the mean of the two natural logarithms of the fat measurements (Figure 5b). There was no significant correlation between the differences and the mean of the liver fat logarithms (Spearman’s rank order correlation coefficient rho = 0.1051, p = 0.4283). The mean difference between the measurements (−0.103, standard deviation = 0.495) was not significantly different from 0 (p = 0.1167). Furthermore, the mean differences between measurements for each of the three different scanners used in the study were not significantly different from zero (p = 0.65, 0.12, and 0.32). The upper and lower 95% limits of agreement between the two logarithms of fat measurements were found to be −1.073 (95% CI −1.296 to −0.85) and 0.868 (95% CI 0.645–1.091), respectively (Figure 5b). These limits of agreement correspond to 95% of pairs of liver fat measurements being expected to have ratios (fat fraction measured by morphometry to fat fraction measured by MRI) between 0.342 (95% CI 0.274–0.427) and 2.382 (95% CI 1.907–2.976).

## Discussion

In this cohort of patients with heterogenous liver disease, a simple, rapid, quantitative MRI measurement based on in-phase and opposed-phase imaging can provide clinically relevant diagnostic information on liver fat levels. The MRI technique demonstrated high levels of sensitivity and specificity at all clinically relevant thresholds of liver fat (≥5%, >33%, >66%) [2], using the gold standard of liver fat estimated by a pathologist from a biopsy sample. At these three cut-offs we achieved areas under ROC curves of 0.962, 0.993, and 0.972, respectively. Our results compare favourably to the small number of studies that have used a pathologist’s visual estimate of fat content as the reference standard to define thresholds and assess the diagnostic performance of comparable MRI approaches [7], [9], [15], [22], [23], [26], [27] (see Table S2 in File S1). The MRI technique presented here has higher areas under ROC curves for all liver fat thresholds described in the literature (Table S2 in File S1), except for two studies [7], [15] investigating liver fat in non-diseased cohorts (potential living donor candidates). At 5% or more fat, our AUC was marginally lower (0.962 versus 0.987) than the result from Joe et al. [7] and, at 30% or more fat, Lee et al. [15] reported an AUC of 0.995 compared to our result of 0.992. For identifying subjects with histological fat ≥5%, McPherson et al. [22] calculated separate areas under ROC curves for subjects with METAVIR fibrosis stage 1 and below (AUC = 0.97) and subjects with METAVIR fibrosis stage 2 and above (AUC = 0.87). When analysed on a similar basis, our data gave AUCs of 0.95 and 0.99 respectively.

The diagnostic performance of the MRI technique was largely independent of the method of quantifying the fat content in biopsy specimens, consistent with the strong coefficients of determination between α, HIS-VIS, and HIS-MORPH. The AUCs based on thresholds from the morphometric image analysis were similar to those based on the visual cut-offs (Table 2, 3). To our knowledge, no other study has used reference thresholds based on morphometric image analysis to assess the diagnostic performance of in-phase, opposed-phase MRI. The strong diagnostic performance of the MRI method using two independent reference standards suggests that it is robust. In our approach, the difference in T_1_ between fat and tissue water is exploited, by use of a high flip angle, to distort the relationship between fat volume fraction and α away from a linear 1∶1 relationship to a non-linear relationship (Figure 3). For tissues with a limited range of fat volume fraction, *f*, (e.g. from 0.00 to 0.40) the sensitivity of the MRI measurement α for predicting the fraction of protons in fat is potentially enhanced for two reasons. Firstly, the differential of the relationship between the MRI measurement α and the fraction of tissue protons in fat is increased and secondly, the random error on α can be decreased owing to better signal to noise ratios in the MRI data. The results from our study therefore indicate that the particular adaptation of the Dixon technique we used can be a useful non-invasive tool for diagnosing liver fat across a range of liver diseases with high sensitivity and specificity at all grades of steatosis.

This study also showed that the MRI α measurement was strongly associated with visual (r^2^ = 0.83) and computer-assisted morphometric (r^2^ = 0.84) estimates of liver fat from histological specimens. In comparison, several studies have also reported mostly strong correlations between MRI measurements and estimates or grades of liver fat from histology specimens [10]–[12], [15], [17]–[19], [21]–[26], [29], [35], [36]. These studies vary in the types and numbers of patients and the MRI acquisition and processing used (Table S3 in File S1), possibly explaining the large range in reported coefficients of determination (0.26–0.89). Our MRI versus biopsy results showed stronger coefficients of determination than all but two of these studies [17], [36]. However, our study was larger and investigated a broader group of aetiologies compared to these studies (Table S3 in File S1, [17], [36]). The strong coefficients of determination we observed between MRI and histological fat measurements suggest that our data could form the basis of a calibration between a quantitative MRI measurement sensitive to liver fat content and histological estimates of fat.

The Bland Altman analysis summarized in Figure 5 gives an indication of the precision of the MRI measurements for measurement of liver fat insofar as the 95% limits of agreement are a measure of the scatter of the data about the calibration curve. Although some of the scatter will be attributable to sampling and analytical errors in the biopsy measurement, these 95% limits of agreement will enable comparison of the precision of other techniques when compared against the biopsy method in the future. While a separate prospective study with different scanners and a different cohort of subjects will be required to validate the accuracy of the technique for general use, there was no detectable bias in the calibration between the three scanners used in this study.

Our study has some limitations. Subjects did not have MRI and liver biopsy performed on the same day with potential for changes in hepatic steatosis levels between assessments. However, the strong associations and diagnostic performance we observed suggest that any changes in fat levels between biopsy and imaging were not great. While our cohort size was larger than many comparable studies, further validation studies on a larger independent patient group would be desirable. In-phase, opposed-phase MRI methods can potentially give erroneous liver fat estimates when the ratio of fat to water protons in the liver exceeds 0.5, leading to water/fat ambiguity issues [33]. In this study we assumed that the number of water protons in the liver always exceeded the number of fat protons. We made this assumption based on the results of the Dallas Heart Study [37], which reported that none of the 2287 subjects they studied with MRS exceeded 50% hepatic triglyceride content. Compared with MRS methods, whole liver imaging methods such as the one reported here are unable to give detailed spectroscopic information on the fat content of the liver.

In summary, we have shown that a rapid (single breath-hold), routinely available, triple gradient echo MRI method has a high degree of sensitivity and specificity to the amount of liver fat measured in a biopsy specimen, either by a pathologist’s visual estimate or by morphometric image analysis. Furthermore, this study indicates that this MRI approach provides noninvasive measurement of liver fat with high sensitivity and specificity for diagnosing liver fat at all grades of steatosis in a cohort with a range of liver conditions.

## Supporting Information

File S1 **Table S1.** Data reported in this study. **Table S2.** Summary of studies reporting diagnostic performance of comparable MRI approaches using a pathologist’s visual estimate of fat content as the reference standard. **Table S3.** Summary of studies comparing histologic fat estimates to MRI.
(DOC)

## References

[1] Reeder SB, Sirlin CB (2010) Quantification of Liver Fat with Magnetic Resonance Imaging. Magnetic resonance imaging clinics of North America 18: 337. 21094444 10.1016/j.mric.2010.08.013 PMC3002753

[2] Kleiner DE, Brunt EM, Van Natta M, Behling C, Contos MJ, et al (2005) Design and validation of a histological scoring system for nonalcoholic fatty liver disease. Hepatology 41: 1313–1321. 15915461 10.1002/hep.20701

[3] El Badry AM (2009) Assessment of hepatic steatosis by expert pathologists: the end of a gold standard. Annals of surgery 250: 691. 19806055 10.1097/SLA.0b013e3181bcd6dd

[4] Siegel CA, Silas AM, Suriawinata AA, van Leeuwen DJ (2005) Liver biopsy 2005: when and how? Cleveland Clinic Journal of Medicine 72: 199–201. 15825800 10.3949/ccjm.72.3.199

[5] Dixon WT (1984) Simple proton spectroscopic imaging. Radiology 153: 189–194. 6089263 10.1148/radiology.153.1.6089263

[6] Brix G, Heiland S, Bellemann ME, Koch T, Lorenz WJ (1993) MR imaging of fat-containing tissues: Valuation of two quantitative imaging techniques in comparison with localized proton spectroscopy. Magnetic Resonance Imaging 11: 977–991. 8231682 10.1016/0730-725x(93)90217-2

[7] Joe E, Lee JM, Kim KW, Lee KB, Kim SJ, et al (2012) Quantification of hepatic macrosteatosis in living, related liver donors using T1-independent, T2*-corrected chemical shift MRI. Journal of Magnetic Resonance Imaging 36: 1124–1130. 22761083 10.1002/jmri.23738

[8] Kühn J-P, Evert M, Friedrich N, Kannengiesser S, Mayerle J, et al (2011) Noninvasive Quantification of Hepatic Fat Content Using Three-Echo Dixon Magnetic Resonance Imaging With Correction for T2* Relaxation Effects. Investigative Radiology 46: 783–789. 21808200 10.1097/RLI.0b013e31822b124c

[9] Kang B-K, Yu ES, Lee SS, Lee Y, Kim N, et al (2012) Hepatic Fat Quantification: A Prospective Comparison of Magnetic Resonance Spectroscopy and Analysis Methods for Chemical-Shift Gradient Echo Magnetic Resonance Imaging With Histologic Assessment as the Reference Standard. Investigative Radiology 47: 368–375. 22543969 10.1097/RLI.0b013e31824baff3

[10] Bahl M, Qayyum A, Westphalen AC, Noworolski SM, Chu PW, et al (2008) Liver Steatosis: Investigation of Opposed-Phase T1-weighted Liver MR Signal Intensity Loss and Visceral Fat Measurement as Biomarkers1. Radiology 249: 160–166. 18796674 10.1148/radiol.2491071375 PMC2657853

[11] Westphalen ACA, Qayyum A, Yeh BM, Merriman RB, Lee JA, et al (2007) Liver Fat: Effect of Hepatic Iron Deposition on Evaluation with Opposed-Phase MR Imaging. Radiology 242: 450–455. 17255416 10.1148/radiol.2422052024

[12] Fishbein M, Castro F, Cheruku S, Jain S, Webb B, et al (2005) Hepatic MRI for Fat Quantitation: Its Relationship to Fat Morphology, Diagnosis, and Ultrasound. Journal of Clinical Gastroenterology 39: 619–625. 16000931 10.1097/00004836-200508000-00012

[13] Thomsen C, Becker U, Winkler K, Christoffersen P, Jensen M, et al (1994) Quantification of liver fat using magnetic resonance spectroscopy. Magnetic Resonance Imaging 12: 487–495. 8007779 10.1016/0730-725x(94)92543-7

[14] Longo R, Pollesello P, Ricci C, Masutti F, Kvam BJ, et al (1995) Proton MR spectroscopy in quantitative in vivo determination of fat content in human liver steatosis. Journal of Magnetic Resonance Imaging 5: 281–285. 7633104 10.1002/jmri.1880050311

[15] Lee SS, Park SH, Kim HJ, Kim SY, Kim M-Y, et al (2010) Non-invasive assessment of hepatic steatosis: Prospective comparison of the accuracy of imaging examinations. Journal of Hepatology 52: 579–585. 20185194 10.1016/j.jhep.2010.01.008

[16] Hussain HK, Chenevert TL, Londy FJ, Gulani V, Swanson SD, et al (2005) Hepatic Fat Fraction: MR Imaging for Quantitative Measurement and Display - Early Experience. Radiology 237: 1048–1055. 16237138 10.1148/radiol.2373041639

[17] Cowin GJ, Jonsson JR, Bauer JD, Ash S, Ali A, et al (2008) Magnetic resonance imaging and spectroscopy for monitoring liver steatosis. Journal of Magnetic Resonance Imaging 28: 937–945. 18821619 10.1002/jmri.21542

[18] d’Assignies G, Ruel M, Khiat A, Lepanto L, Chagnon M, et al (2009) Noninvasive quantitation of human liver steatosis using magnetic resonance and bioassay methods. European Radiology 19: 2033–2040. 19280194 10.1007/s00330-009-1351-4

[19] d’Assignies G, Kauffmann C, Boulanger Y, Bilodeau M, Vilgrain V, et al (2010) Simultaneous assessment of liver volume and whole liver fat content: a step towards one-stop shop preoperative MRI protocol. European Radiology 21: 301–309. 20814683 10.1007/s00330-010-1941-1

[20] Roldan-Valadez E, Favila R, Martinez-Lopez M, Uribe M, Rios C, et al (2010) In vivo 3T spectroscopic quantification of liver fat content in nonalcoholic fatty liver disease: Correlation with biochemical method and morphometry. Journal of Hepatology 53: 732–737. 20594607 10.1016/j.jhep.2010.04.018

[21] Hatta T, Fujinaga Y, Kadoya M, Ueda H, Murayama H, et al (2010) Accurate and simple method for quantification of hepatic fat content using magnetic resonance imaging: a prospective study in biopsy-proven nonalcoholic fatty liver disease. Journal of Gastroenterology 45: 1263–1271. 20625773 10.1007/s00535-010-0277-6

[22] McPherson S, Jonsson JR, Cowin GJ, O'Rourke P, Clouston AD, et al (2009) Magnetic resonance imaging and spectroscopy accurately estimate the severity of steatosis provided the stage of fibrosis is considered. Journal of Hepatology 51: 389–397. 19505740 10.1016/j.jhep.2009.04.012

[23] Pilleul F, Chave G, Dumortier J, Scoazec J, Valette P (2005) Detection and grading using dual T1 gradient echo sequences on clinical MR system. Gastroentérologie clinique et biologique 29: 1143. 16505760 10.1016/s0399-8320(05)82179-7

[24] Qayyum A, Goh JS, Kakar S, Yeh BM, Merriman RB, et al (2005) Accuracy of Liver Fat Quantification at MR Imaging: Comparison of Out-of-Phase Gradient-Echo and Fat-saturated Fast Spin-Echo Techniques - Initial Experience. Radiology 237: 507–511. 16244259 10.1148/radiol.2372040539

[25] Rinella ME, McCarthy R, Thakrar K, Finn JP, Rao SM, et al (2003) Dual-echo, chemical shift gradient-echo magnetic resonance imaging to quantify hepatic steatosis: Implications for living liver donation. Liver Transplantation 9: 851–856. 12884199 10.1053/jlts.2003.50153

[26] Mennesson N, Dumortier J, Hervieu V, Milot L, Guillaud O, et al (2009) Liver steatosis quantification using magnetic resonance imaging: a prospective comparative study with liver biopsy. Journal of Computer Assisted Tomography 33: 672. 19820490 10.1097/RCT.0b013e318199d883

[27] Cho CS, Curran S, Schwartz LH, Kooby DA, Klimstra DS, et al (2008) Preoperative Radiographic Assessment of Hepatic Steatosis with Histologic Correlation. Journal of the American College of Surgeons 206: 480–488. 18308219 10.1016/j.jamcollsurg.2007.08.020

[28] Brunt EM, Janney CG, Di Bisceglie AM, Neuschwander-Tetri BA, Bacon BR (1999) Nonalcoholic steatohepatitis: a proposal for grading and staging the histological lesions. The American Journal of Gastroenterology 94: 2467–2474. 10484010 10.1111/j.1572-0241.1999.01377.x

[29] Cesbron-Métivier E, Roullier V, Boursier J, Cavaro-Ménard C, Lebigot J, et al (2010) Noninvasive liver steatosis quantification using MRI techniques combined with blood markers. European journal of gastroenterology & hepatology 22: 973. 20665947 10.1097/meg.0b013e32833775fb

[30] St Pierre TG, Clark PR, Chua-Anusorn W (2004) Single spin-echo proton transverse relaxometry of iron-loaded liver. NMR in Biomedicine 17: 446–458. 15523601 10.1002/nbm.905

[31] St Pierre TG, Clark PR, Chua-anusorn W, Fleming AJ, Jeffrey GP, et al (2005) Noninvasive measurement and imaging of liver iron concentrations using proton magnetic resonance. Blood 105: 855–861. 15256427 10.1182/blood-2004-01-0177

[32] St Pierre TG, El-Beshlawy A, Elalfy MS, Al Jefri A, Al Zir K, et al (2010) Multicenter Validation of Spin-Density Projection-Assisted R2-MRI for the Non-Invasive Measurement of Liver Iron Concentration. Blood 116: 852–852. 23821350 10.1002/mrm.24854 PMC4238736

[33] Bydder M, Yokoo T, Hamilton G, Middleton MS, Chavez AD, et al (2008) Relaxation effects in the quantification of fat using gradient echo imaging. Magnetic Resonance Imaging 26: 347–359. 18093781 10.1016/j.mri.2007.08.012 PMC2386876

[34] Bland JM, Altman DG (1999) Measuring agreement in method comparison studies. Statistical Methods in Medical Research 8: 135–160. 10501650 10.1177/096228029900800204

[35] Kim SH, Lee JM, Han JK, Lee JY, Lee KH, et al (2006) Hepatic Macrosteatosis: Predicting Appropriateness of Liver Donation by Using MR Imaging - Correlation with Histopathologic Findings. Radiology 240: 116–129. 16684918 10.1148/radiol.2393042218

[36] Schuchmann S, Weigel C, Albrecht L, Kirsch M, Lemke A, et al (2007) Non-invasive quantification of hepatic fat fraction by fast 1.0, 1.5 and 3.0T MR imaging. European Journal of Radiology 62: 416–422. 17267159 10.1016/j.ejrad.2006.12.009

[37] Szczepaniak LS, Nurenberg P, Leonard D, Browning JD, Reingold JS, et al (2005) Magnetic resonance spectroscopy to measure hepatic triglyceride content: prevalence of hepatic steatosis in the general population. American Journal of Physiology - Endocrinology And Metabolism 288: E462–E468. 15339742 10.1152/ajpendo.00064.2004

