# Supplementary material for: Diagnostic Performance of a Rapid Magnetic Resonance Imaging Method of Measuring Hepatic Steatosis
Source: PLoS One. 2013 Mar 21;8(3):e59287. doi: 10.1371/journal.pone.0059287 (PMC3605443; doi:10.1371/journal.pone.0059287)
Supplement: File S1 — Table S1. Data reported in this study. Table S2. Summary of studies reporting diagnostic performance of comparable MRI approaches using a pathologist’s visual estimate of fat content as the reference standard. Table S3. Summary of studies comparing histologic fat estimates to MRI. (DOC) [file pone.0059287.s001.doc]

**Table S1. Data reported in this study.**

| ID | AGE at MRI | GENDER | AETIOLOGY | BMI | LIC (mg/g dry tissue) | METAVIR | MRI-BIOPSY (DAYS) | HIST-VIS % | HIST-MORPH | Alpha | Comment |
| --- | --- | --- | --- | --- | --- | --- | --- | --- | --- | --- | --- |
| C001 | 44 | M | CON | 23.5 | 0.9 | N/A | N/A | N/A | N/A | 0.013 |  |
| C002 | 42 | M | CON | 19.3 | 1.8 | N/A | N/A | N/A | N/A | 0.015 |  |
| C003 | 26 | M | CON | 24.8 | 1.5 | N/A | N/A | N/A | N/A | 0.018 |  |
| C004 | 24 | F | CON | 23.3 | 0.7 | N/A | N/A | N/A | N/A | 0.023 |  |
| C005 | 47 | M | CON | 21.9 | 1.3 | N/A | N/A | N/A | N/A | 0.032 |  |
| C006 | 36 | M | CON | 24.0 | 0.4 | N/A | N/A | N/A | N/A | 0.105 |  |
| C007 | 32 | F | CON | 19.8 | 1.1 | N/A | N/A | N/A | N/A | 0.057 |  |
| C008 | 31 | M | CON | 23.1 | 1.4 | N/A | N/A | N/A | N/A | 0.025 |  |
| C009 | 30 | F | CON | 23.1 | 0.6 | N/A | N/A | N/A | N/A | 0.023 |  |
| C010 | 47 | M | CON | 22.6 | 1.2 | N/A | N/A | N/A | N/A | 0.027 |  |
| NF001 | 57 | F | NASH | 35.9 | 0.6 | F2 | 56 | 80% | 0.097 | 0.247 |  |
| NF002 | 22 | M | PSC | 27.4 | 1.0 | F3 | 59 | 0% | 0.007 | 0.026 |  |
| NF003 | 51 | M | HCV | 22.7 | 1.1 | F4 | 3 | <1% | 0.006 | 0.025 |  |
| NF004 | 56 | M | CIRR | 25.7 | 0.7 | F4 | 460 | 0% | 0.006 | 0.026 |  |
| NF005 | 59 | M | NAFLD | 26.3 | 1.1 | F1 | 79 | 98% | 0.062 | 0.207 |  |
| NF006 | 40 | F | HBV-HCV | 20.8 | 0.6 | F2 | 8 | 0% | 0.008 | 0.017 |  |
| NF007 | 51 | F | HCV / HH | 26.4 | 0.5 | F2 | 45 | 1% | 0.007 | 0.049 |  |
| NF008 | 56 | M | NORM | 23.7 | 0.9 | F0 | 69 | <0.5% | 0.002 | 0.026 |  |
| NF009 | 20 | F | PSC | 25.4 | 0.9 | F3 | 50 | 0% | 0.010 | 0.013 |  |
| NF010 | 62 | F | AIH | 25.4 | 0.5 | F4 | 158 | 5% | 0.015 | 0.047 |  |
| NF011 | 54 | M | HCV | 26.6 | 0.8 | F4 | 9 | 90% | 0.033 | 0.176 |  |
| NF012 | 61 | F | HBV | 23.1 | 1.3 | F3 | 232 | 90% | 0.090 | 0.207 |  |
| NF013 | 26 | M | NASH | 31.1 | 1.0 | F0 | 171 | 50% | 0.054 | 0.217 |  |
| NF014 | 65 | M | HCV | 23.7 | 0.7 | F1 | 50 | 90% | 0.037 | 0.188 |  |
| NF015 | 56 | F | HCV | 26.0 | 0.7 | F3 | -19 | 0% | 0.005 | 0.014 |  |
| NF016 | 37 | F | HBV | 19.2 | 1.0 | F1 | 15 | <1% | 0.010 | 0.026 |  |
| NF017 | 49 | F | HBV | 28.5 | 1.0 | F3 | 152 | 0% | 0.005 | 0.024 |  |
| NF018 | 63 | M | HEM | 30.4 | 3.3 | F1 | -2 | 60% | 0.058 | 0.163 |  |
| NF019 | 49 | F | HBV | 31.4 | 1.5 | F2 | 73 | 0% | 0.003 | 0.016 |  |
| NF020 | 63 | M | ALD | 30.3 | 4.8 | F3-4 | -58 | 60% | 0.036 |  | Excluded - incorrect MRI sequence |
| NF021 | 62 | M | AIH | 30.5 | 0.5 | F4 | -1 | 1% | 0.008 | 0.101 |  |
| NF022 | 48 | F | Drug-induced liver injury | 29.0 | 2.3 | F0 | 298 | <0.5% | 0.006 | 0.022 |  |
| NF023 | 20 | M | PSC | 22.0 | 0.5 | F3 | 44 | 0% | 0.006 | 0.016 |  |
| NF024 | 64 | F | NASH | 26.3 | 1.3 | F1 | -19 | 30% | 0.077 | 0.188 |  |
| NF025 | 62 | F | NAFLD | 32.8 | 1.4 | F0 | 223 | 25% | 0.032 | 0.126 |  |
| NF026 | 67 | F | NAFLD | 33.1 | 0.7 | F0 | 239 | <0.5% | 0.007 | 0.029 |  |
| NF027 | 69 | M | ALD | 29.5 | 4.4 | F1 | 36 | <1% | 0.005 | 0.036 |  |
| NF028 | 39 | M | NASH | 33.3 | 0.4 | F0 | 50 | 40% | 0.041 | 0.165 |  |
| NF029 | 55 | M | NASH | 31.6 | 0.6 | F1 | 157 | 70% | 0.105 | 0.198 |  |
| NF030 | 64 | M | NAFLD | 28.7 | 1.3 | F0 | 13 | 80% | 0.108 | 0.241 |  |
| NF031 | 46 | F | NORM | 22.2 | 1.3 | F0 | 76 | 0% | 0.005 | 0.022 |  |
| NF032 | 43 | M | NORM | 32.4 | 1.4 | F0 | 306 | 4% | 0.030 | 0.063 |  |
| NF033 | 56 | M | NAFLD | 33.5 | 1.5 | F0 | 58 | <0.5% | 0.009 | 0.032 |  |
| NF034 | 36 | M | NASH | 30.2 | 0.6 | F0 | 113 | 80% | 0.097 | 0.309 |  |
| NF035 | 56 | F | NASH | 38.2 | 0.8 | F1 | 72 | 20% | 0.029 | 0.132 |  |
| NF036 | 22 | M | NASH | 18.4 | 0.9 | F1 | 16 | 3% | 0.008 | 0.023 |  |
| NF037 | 56 | F | NASH | 28.1 | 1.8 | F2 | 57 | 95% | 0.088 | 0.219 |  |
| NF038 | 40 | M | PSC | 32.3 | 1.0 | F3 | 39 | 20% | 0.029 | 0.110 |  |
| NF039 | 64 | F | NASH | 37.8 | 0.8 | F0 | 68 | 99% | 0.167 |  | Excluded - incorrect MRI sequence |
| NF040 | 50 | M | HCV | 29.9 | 1.2 | F1 | 48 | 5% | 0.011 | 0.058 |  |
| NF041 | 60 | M | HCV | 27.3 | 1.4 | F1 | 198 | 0% | 0.007 | 0.029 |  |
| NF042 | 57 | F | HCV | 23.6 | 0.6 | F3 | 20 | 0% | 0.011 | 0.024 |  |
| NF043 | 56 | M | HCV | 25.2 | 0.7 | F1 | 94 | 10% |  | 0.076 | Excluded - image scan of biopsy not available |
| NF044 | 72 | F | NAFLD | 27.6 | 1.7 | F3 | 29 | 95% | 0.088 | 0.289 |  |
| NF045 | 54 | M | NASH | 32.4 | 1.8 | F1 | 146 | 70% | 0.121 | 0.126 | Excluded - medical history |
| NF046 | 72 | F | NAFLD | 45.7 | 1.0 | F0 | 162 | 30% | 0.039 | 0.118 |  |
| NF047 | 60 | F | NAFLD | 30.1 | 1.0 | F0 | 142 | 30% | 0.041 | 0.072 |  |
| NF048 | 42 | F | NAFLD | 29.1 | 0.5 | F0 | 44 | 40% | 0.084 | 0.227 |  |
| NF049 | 54 | M | HCV | 24.7 | 0.6 | F3 | 61 | 40% |  | 0.184 | Excluded - image scan of biopsy not available |
| NF050 | 46 | F | ALD | 35.3 | 0.6 | F4 | 38 | 0% | 0.008 | 0.014 |  |
| NF051 | 41 | M | HBV | 22.0 | 0.5 | F1 | 117 | 5% | 0.009 | 0.011 |  |
| NF052 | 39 | M | HBV | 21.0 | 2.0 | F3 | 107 | 2% | 0.005 | 0.030 |  |
| NF053 | 62 | F | PBC | 26.2 | 0.9 | F3 | 23 | <1% | 0.017 | 0.026 |  |
| NF054 | 53 | F | NAFLD | 28.2 | 1.1 | F0 | 430 | 60% |  | 0.137 | Excluded - image scan of biopsy not available |
| NF055 | 43 | M | HCV | 28.7 | 0.5 | F1 | 115 | 4% | 0.023 | 0.025 |  |
| NF056 | 69 | F | AIH | 31.6 | 0.8 | F2 | 135 | Not read | 0.010 | 0.034 |  |
| NF057 | 60 | M | NAFLD | 29.4 | 0.6 | F0 | 47 | Not read | 0.075 | 0.150 |  |
| NF058 | 41 | M | NASH | 35.3 | 0.6 | F1 | 106 | Not read | 0.216 | 0.346 |  |
| NF059 | 49 | M | NASH | 27.7 | 2.7 | F1 | 39 | 80% | 0.101 | 0.326 |  |
| NF060 | 60 | F | NASH | 39.8 | 0.6 | F4 | -2 | 45% | 0.063 | 0.198 |  |
| NF061 | 65 | F | NASH | 30.2 | 0.3 | F1 | 62 | 95% | 0.152 | 0.409 |  |
| NF062 | 61 | F | NASH | 32.3 | 0.6 | F2 | 51 | 70% | 0.109 | 0.239 |  |
| NF063 | 62 | M | NASH | 33.6 | 0.9 | F0 | 28 | 80% | 0.181 | 0.277 |  |
| NF064 | 58 | F | NASH | 34.9 | 1.3 | F3 | 113 | 75% | 0.084 | 0.203 |  |
| NF065 | 39 | M | NASH | 33.1 | 0.5 | F3 | 100 | 85% | 0.079 | 0.344 |  |

Abbreviations: AIH, autoimmune hepatitis; ALD, alcoholic liver disease; BMI, body mass index; CIRR, cirrhosis; CON, control; HBV-HCV, viral hepatitis B/C; HEM, haemochromatosis; LIC, liver iron concentration; MRI, magnetic resonance imaging; NAFLD, non-alcoholic fatty liver disease; NASH, nonalcoholic steatohepatitis; NORM, normal; PBC, primary biliary cirrhosis; PSC, primary sclerosing cholangitis.

**Table S2.** **Summary of studies reporting diagnostic performance of comparable MRI approaches using a pathologist’s visual estimate of fat content as the reference standard.**

| **Study** | **Hepatic Fat**  **Cut-off** | **AUC** | **Sensitivity** | **Specificity** |
| --- | --- | --- | --- | --- |
| This Study | ≥ 5% | 0.962 | 90.9 | 96.2 |
| Joe 2012 | ≥ 5% | 0.987 | 87.5 | 97.0 |
| Kang 2012 | ≥ 5% | 0.947 | 100 | 78.9 |
| Mennesson 2009 | ≥ 5% | 0.945* | 96.9 | 85.7 |
| Lee 2010 | ≥ 5% | 0.883 | 76.7 | 87.1 |
| Cho 2008 | Any steatosis |  | 57.0 | 64.0 |
|  |  |  |  |  |
| This Study | ≥ 5% (MV0-1) | 0.948 | 90.5 | 100 |
| McPherson 2009 | ≥ 5% (MV0-1) | 0.970 | 88.0 | 100 |
| This Study | ≥ 5% (MV2-4) | 0.989 | 91.7 | 100 |
| McPherson 2009 | ≥ 5% (MV2-4) | 0.870 | 77.0 | 83.0 |
|  |  |  |  |  |
| This Study | > 10% | 0.998 | 100 | 96.6 |
| Pilleul 2005 | > 10% |  | 80.0 | 71.0 |
|  |  |  |  |  |
| This Study | ≥ 20% | 0.998 | 96.6 | 100 |
| Mennesson 2009 | ≥ 20% | 0.972* | 96.0 | 92.9 |
|  |  |  |  |  |
| This Study | ≥ 30% | 0.992 | 92.3 | 100 |
| Lee 2010 | ≥ 30% | 0.995 | 90.9 | 94.0 |
| Cho 2008 | ≥ 30% |  | 88.0 | 63.0 |
|  |  |  |  |  |
| This Study | > 33% | 0.993 | 100 | 97.22 |
| This Study | > 33% (MV0-1) | 0.984 | 100 | 94.74 |
| McPherson 2009 | > 33% (MV0-1) | 0.980 | 93.0 | 97.0 |
| This Study | > 33% (MV2-4) | 1.00 | 100 | 100 |
| McPherson 2009 | > 33% (MV2-4) | 0.890 | 85.0 | 97.0 |
| This Study | > 66% | 0.972 | 100 | 88.4 |

Abbreviations: AUC – Area under receiver operating characteristic curve; MV – METAVIR

* As calculated from publication data.

**Table S3.** **Summary of studies comparing histologic fat estimates to MRI.**

| **Reference** | **Patients (n)** | **Aetiology** | **Histology Measurement** | **r^2^**  **HIS-VIS vs MRI** | **r^2^**  **HIS-MORPH vs MRI** | **Field T** |
| --- | --- | --- | --- | --- | --- | --- |
| This Study | 59 | NAFLD, NASH, HBV, HCV, PSC | VC, AOSeg | 0.83 | 0.84 | 1.5 |
| Bahl 2008 | 52 | NAFLD, HCV, HIV | VG | 0.61 |  | 1.5 |
| Cesbron-Métivier 2010 | 23 | NAFLD | VG, AOSeg | 0.61 | 0.52 | 1.5 |
| Cowin 2008 | 12 | NAFLD, HCV | VC | 0.89 |  | 1.5 |
| d’Assignies 2009 | 20 | NAFLD, AFLD | VC, AOSeg | 0.26 | 0.55 | 1.5 |
| Fishbein 2005 | 38 | HCV, NAFLD, AIH, other | VG | 0.59 |  | 1.5 |
| Hatta 2010 | 26 | NAFLD | MOSeg |  | 0.82 | 1.5 |
| Joe 2012 | 49 | No liver disease (potential living donor) | VC | 0.75 |  | 1.5 |
| Kang 2012 | 56 | HCN, HBV, HCV | VC | 0.81 |  | 1.5 |
| Kim 2006 | 57 | 50 potential living donor, 7 NAFLD | VC | 0.72 |  | 1.5 |
| Kuhn 2011 | 100 | Cancer patients | VC | 0.79 |  | 1.5 |
| Lee 2010 | 161 | No liver disease (potential living donor) | VC | 0.71 |  | 3 |
| Mennesson 2009 | 40 | NAFLD, ASH, PSC, AIH | VC | 0.73 |  | 1.5 |
| McPherson 2009 | 92 | HCV, HBV, NAFLD, AFLD, AIH | VG | 0.77 |  | 1.5 |
| Pilleul 2005 | 25 | hepatitis, focal lesions | VC | 0.67 |  | 1.5 |
| Qayyum 2005 | 11 | CIRR, HCV | VC | 0.48 |  | 1.5 |
| Rinella 2003 | 22 | 15 potential living donor, 7 NAFLD | VG | 0.71 |  | 1.5 |
| Schuchmann 2007 | 24 | MG, HCN | VC | 0.86 |  | 1.0 |
| Westphalen 2007 | 13 | HCV, HBV, CIRR, PSC | VC | 0.49 |  | 1.5 |

Abbreviations: AIH – autoimmune hepatitis, AFLD - alcoholic fatty liver disease, AOSeg – automatic optical segmentation to measure fat area, ASH - alcoholic steatohepatitis, CIRR – cirrhosis, HBV – viral hepatitis B, HCN – hepatocellular carcinoma, HCV viral hepatitis C, HIV – human immuno virus, MG – metastatic growth, MOSeg – manual optical segmentation to measure fat area, NAFLD – non-alcoholic fatty liver disease, NASH non-alcoholic steatohepatitis, PSC – primary sclerosing cholangitis, VC – visual estimate of fat on a continuous scale, VG - visual estimate of fat graded into stages.
